# A116 OPTIMIZING CROHN’S DISEASE ENDOSCOPIC SCORING OPERATING CHARACTERISTICS TO ASSIST WITH THE ADVENT OF ARTIFICIAL INTELLIGENCE

**DOI:** 10.1093/jcag/gwac036.116

**Published:** 2023-03-07

**Authors:** C Galts, N Narula, E Wong, W Reinisch

**Affiliations:** 1 gastroenterology; 2 mcmaster university, hamilton, Canada; 3 Medical University of Vienna, Vienna, Austria

## Abstract

**Background:**

To date, no studies have used Artificial Intelligence (AI) to produce standard endoscopic scores for Crohn’s Disease (CD). These scores are essential to clinical practice and have a major cost in clinical trials.

**Purpose:**

We aimed to re-examine the components of the Simple Endoscopic Score for Crohn’s Disease (SES-CD) and Modified Multiplier (MM-SES-CD) to see if scoring of individual colonic segments could be combined while maintaining predictive value accuracy, thus simplifying scoring for AI.

**Method:**

Data from 279 participants in the UNITI and EXTEND trials as well as an infliximab biosimilar trial (NCT02096861) were used in this retrospective study. The primary outcome was endoscopic remission (ER-1) of the colon, defined as absence of ulcers. Secondary outcomes included alternative definitions of colonic endoscopic remission including SES-CD score of 0 (ER-2), SES-CD <3 (ER-3), and SES-CD reduction by 50% or greater from baseline (colonic endoscopic response).

**Result(s):**

The mean baseline SES-CD score was 13.7 (SD 7.7) and the mean MM-SES-CD score was 22.8 (SD 12.4). Among all possible colonic segment combinations, the combination of Right + Rectum + worst of Left or Transverse performed best for prediction of one-year ER-1, with fair accuracy (AUC 0.71, 95% CI 0.65-0.78, p < 0.0001). Accuracy of prediction of one-year ER-2 was poor (AUC 0.68, 95% CI 0.62-0.75, p < 0.0001), ER-3 was fair (AUC 0.71, 95% CI 0.65-0.77, p < 0.0001), and prediction of endoscopic response was poor (AUC 0.64, 95% CI 0.56-0.72, p > 0.01). These results were very similar to conventional scoring (without combining any “worst of” segments) with all of the colonic MM-SES-CD scores, with AUCs of 0.70, 0.69, 0.71, and 0.61 for the respective outcomes. Assessing only for the presence of ulcers was as accurate as the full assessment and exclusion of scoring stenosis had minimal impact on scores.

**Image:**

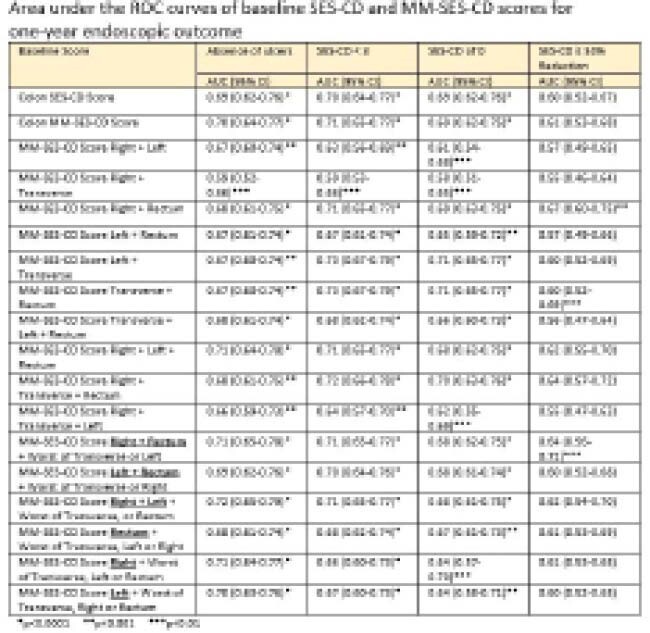

**Conclusion(s):**

By combining assessment of colonic segments accuracy was maintained compared with currently used endoscopic scoring. Assessing for features other than ulceration had no significant impact on overall scores. By combining colonic segments and limiting assessment to ulceration, it may be simpler to develop AI algorithms reliably scoring endoscopic severity of CD for clinical practice and trials.

**Please acknowledge all funding agencies by checking the applicable boxes below:**

None

**Disclosure of Interest:**

None Declared

